# Flexible Neural Electrode Array Based-on Porous Graphene for Cortical Microstimulation and Sensing

**DOI:** 10.1038/srep33526

**Published:** 2016-09-19

**Authors:** Yichen Lu, Hongming Lyu, Andrew G. Richardson, Timothy H. Lucas, Duygu Kuzum

**Affiliations:** 1 Department of Electrical & Computer Engineering, University of California, San Diego, La Jolla, CA 92093, USA; 2 Department of Neurosurgery, Perelman School of Medicine, University of Pennsylvania, Philadelphia, Pennsylvania 19104, USA

## Abstract

Neural sensing and stimulation have been the backbone of neuroscience research, brain-machine interfaces and clinical neuromodulation therapies for decades. To-date, most of the neural stimulation systems have relied on sharp metal microelectrodes with poor electrochemical properties that induce extensive damage to the tissue and significantly degrade the long-term stability of implantable systems. Here, we demonstrate a flexible cortical microelectrode array based on porous graphene, which is capable of efficient electrophysiological sensing and stimulation from the brain surface, without penetrating into the tissue. Porous graphene electrodes show superior impedance and charge injection characteristics making them ideal for high efficiency cortical sensing and stimulation. They exhibit no physical delamination or degradation even after 1 million biphasic stimulation cycles, confirming high endurance. In *in vivo* experiments with rodents, same array is used to sense brain activity patterns with high spatio-temporal resolution and to control leg muscles with high-precision electrical stimulation from the cortical surface. Flexible porous graphene array offers a minimally invasive but high efficiency neuromodulation scheme with potential applications in cortical mapping, brain-computer interfaces, treatment of neurological disorders, where high resolution and simultaneous recording and stimulation of neural activity are crucial.

Intracranial recording and stimulation have played critically important roles in studying basic neural processes underlying human behavior and causes of neurological disorders^1^,^2^. Current clinical practices for recoding and stimulation mostly rely on penetrating deep brain leads with millimeter-scale electrodes^3^,^4^ while brain computer interfaces (BCIs) generally utilize silicon-based sharp microelectrode arrays^5^ implanted in the cortex. Implantation damage, tissue inflammatory response, and corrosion of the electrodes remain as the major issues degrading long-term stability of these penetrating implants. To address these problems, new minimally-invasive technologies involving nanowire FET sensors^6^ and polymer fiber probes^7^ have been developed. However, these technologies still require penetration to the cortical tissue. As an alternative, recording and stimulation from the cortical surface using electrocorticography (ECoG) arrays have been suggested to be more robust and less invasive than using penetrating electrode arrays or probe shanks^8^,^9^,^10^,^11^. ECoG-based stimulation is being adopted in an increasing number of applications, including treatment of seizures, control of remote prostheses, and evoking somatosensory sensations^10^,^11^. Millimeter-scale size and spacing of conventional ECoG arrays limit the resolution for spatiotemporal mapping of neural activity in the cortex^1^,^12^. Robust and flexible microelectrode arrays with highly efficient recording and stimulation capabilities and good cycling endurance can significantly impact the understanding of cortical microcircuits and greatly enhance the spatial resolution for clinical therapies and BCI applications. Consequently, developing low impedance and flexible micro ECoG arrays is an active area of research^13^,^14^,^15^,^16^, while most of these studies focus on recording only.

An ideal cortical microelectrode array for neural recording and stimulation needs to be minimally invasive, flexible and robust, while providing low impedance and high charge transfer capacity. Conventional neural electrodes are made of noble metals and alloys, with platinum electrodes being the most typical ones in clinical use^17^,^18^,^19^,^20^. However, the charge injection capacity (CIC) of platinum is limited below 0.15 mC/cm^2 ^17. In order to increase the charge transfer capacity, a wide range of materials, such as titanium nitride^21^,^22^, iridium oxide^22^,^23^, PEDOT^24^,^25^, Ta_2_O_5_^26^, and carbon nanotube composites have been adopted as the coating layer^27^,^28^,^29^. However, several studies have reported delamination issues during neural stimulation, which could be due to poor adhesion between the coating material and the metal electrode arising from different deposition techniques employed in electrode fabrication steps^23^,^24^.

Graphene has recently become a desirable material for neural applications owing to superior properties such as high conductivity^30^,^31^,^32^, flexibility^30^,^33^, transparency^30^,^34^ and biocompatibility^30^,^35^. Studies involving neural network cultures on graphene-based substrates have shown promising results on graphene’s biocompatibility^35^,^36^. Transparent neural electrodes based on monolayer graphene have been recently demonstrated enabling simultaneous electrophysiology and neuroimaging^37^. Although optical transparency of monolayer graphene is appealing for neuroimaging applications, flat surface of 2D graphene limits charge transfer capacity, significantly impeding the efficiency of microstimulation. Here we present a flexible cortical array for cortical recording and microstimulation based on porous graphene, which is directly grown on polyimide substrate using laser pyrolysis. Laser pyrolysis produces three-dimensional graphene foam^38^,^39^,^40^,^41^,^42^, scalable to large area fabrication. Direct growth on the substrate eliminates the delamination problem associated with the coatings. High density microelectrode arrays built on polyimide substrate exhibit drastically low impedance, high charge injection capacity and flexibility, making them ideal for cortical recording and microstimulation. We have demonstrated in *in vivo* experiments with rodents that low impedance of porous graphene microelectrodes allows recording of low-amplitude evoked potentials from the rat’s somatosensory cortex with high signal-to-noise ratio. High efficiency cortical stimulation in the motor cortex has been shown to evoke transient ankle and knee flexion using same exact arrays in consecutive recording and stimulation experiments.

## Results and Discussion

**Figure 1:**
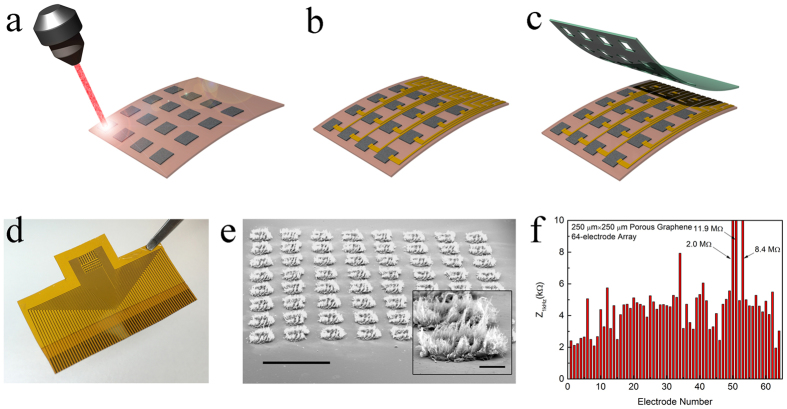
Porous graphene electrode array fabrication. Schematics illustrating (**a**) laser pyrolysis, (**b**) metal interconnects and (**c**) SU-8 encapsulation. (**d**) Photograph of a fabricated 64-electrode array. (**e**) Tilt SEM image of a 64-spot porous graphene array. Scale bar: 1mm. The inset is the SEM image of an individual spot. Scale bar: 100 μm. (**f**) Impedance of all 64 electrodes at 1 kHz.

We fabricated porous graphene arrays using the process flow shown in Fig. 1a–c. Porous graphene spots were first patterned on a polyimide film using direct laser pyrolysis, as illustrated in Fig. 1a. Limited by the spot size of the CO_2_ laser, which is about 130 μm, and the resolution of the software that drives the laser head, direct laser pyrolysis provides porous graphene spots of 250 μm or larger side length with acceptable consistency and uniformity for the lithography afterward. As for patterning smaller structures, an indirect approach with a shadow mask during pyrolysis was also investigated, as shown in Figure S1 in Supplementary Information. 100 nm thick and 25 μm wide Au wires were patterned using e-beam evaporation, photolithography and chemical etching (Fig. 1b). A buffer region of 50 μm width between the wire and the nearest porous graphene spot is necessary to maintain the uniformity of the wires, because the direct laser pyrolysis changes the topography of the polyimide film adjacent to the porous graphene spots. SU8 was deposited and patterned as the encapsulation layer. Figure 1d shows the picture of a fabricated 64-electrode porous graphene array. A tilt SEM image is shown in Fig. 1e, with the inset showing an individual electrode. The SEM images confirm the porous morphology of the electrode surfaces and also show the spatial resolution is 500 μm. With the aforementioned shadow mask method or micropatterning, the spatial resolution can be scaled down to 50 μm. The temporal resolution of the recordings depends on the sampling frequency of the recording system (Intan RHD2000 Evaluation System). We used a sampling frequency of 10 KHz, leading to a temporal resolution of 0.1 ms. The impedance of the 64 electrodes in 0.01 M phosphate-buffered saline (PBS) solution at 1 kHz is shown in Fig. 1f. 61 out of 64 electrodes exhibit impedances within 2 kΩ to 8 kΩ, confirming the uniformity and high yield (95%). Chemical doping technique was adopted, to reduce the sheet resistance of monolayer graphene^43^,^44^. For porous graphene, chemicals can penetrate into the foamy structure and may yield better stability compared to planar graphene. We observed that doping with nitric acid substantially decreases the impedance and increased the charge storage and injection capacity (Figures S3 and S4, Supplementary Information). Impedance of doped samples was found to remain relatively stable in PBS solution over 28 days, implying enhanced long-term stability of chemical doping for porous graphene (Figure S5 in Supplementary Information).

**Figure 2:**
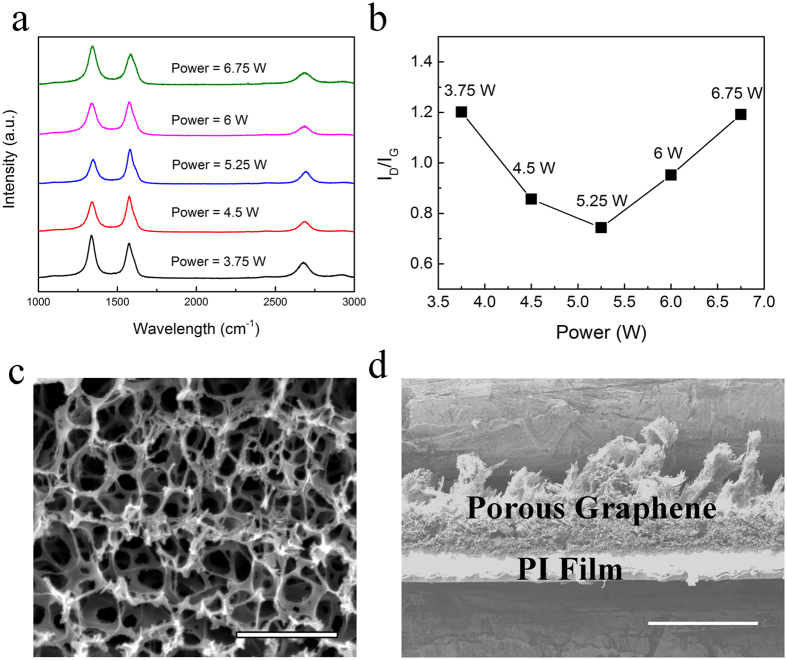
3D porous graphene fabricated by direct laser pyrolysis method. (**a**) Raman spectra of porous graphene samples at different power levels, with I_D_/I_G_ ratio for each sample shown in (**b**). (**c**) SEM image of the porous morphology of surface. Scale bar: 2 μm. (**d**) SEM image of the cross-section view of porous graphene. Scale bar:100 μm.

Porous graphene in various dimensions and geometries can be simply patterned on polyimide substrates using a computer-controlled CO_2_ laser machining system. Besides being flexible, the polyimide substrate provides mechanical support to the porous graphene layer. During pyrolysis on polyimide films, localized temperatures rises over 2500 °C, which breaks the C─C, C**=**O and N─C bonds, as confirmed by the dramatically decreased oxygen and nitrogen contents. Then aromatic compounds rearrange to form graphene structures^31^. Raman spectra of the porous graphene samples acquired at different power levels are shown in Fig. 2a. Three dominant peaks, the G peak at 1580 cm^−1^, the 2D peak at 2700 cm^−1^, and the D peak at 1350 cm^−1^, are observed. The D peak represents defects or bent sp^2^ carbon bonds. The increase in laser power results in a decrease in D peak initially and a subsequent increase with power beyond 5.5 W, as shown by a minimum in the intensity ratio of D and G peaks in Fig. 2b. Figure 2c shows the SEM image of the porous morphology of graphene foam, which indicates pore sizes around 0.2 μm. The porous three-dimensional network leads to large effective surface area, and hence significantly improves the charge injection capacity. Figure 2d shows the cross-section view of a large-area blanket porous graphene film on polyimide.

**Figure 3:**
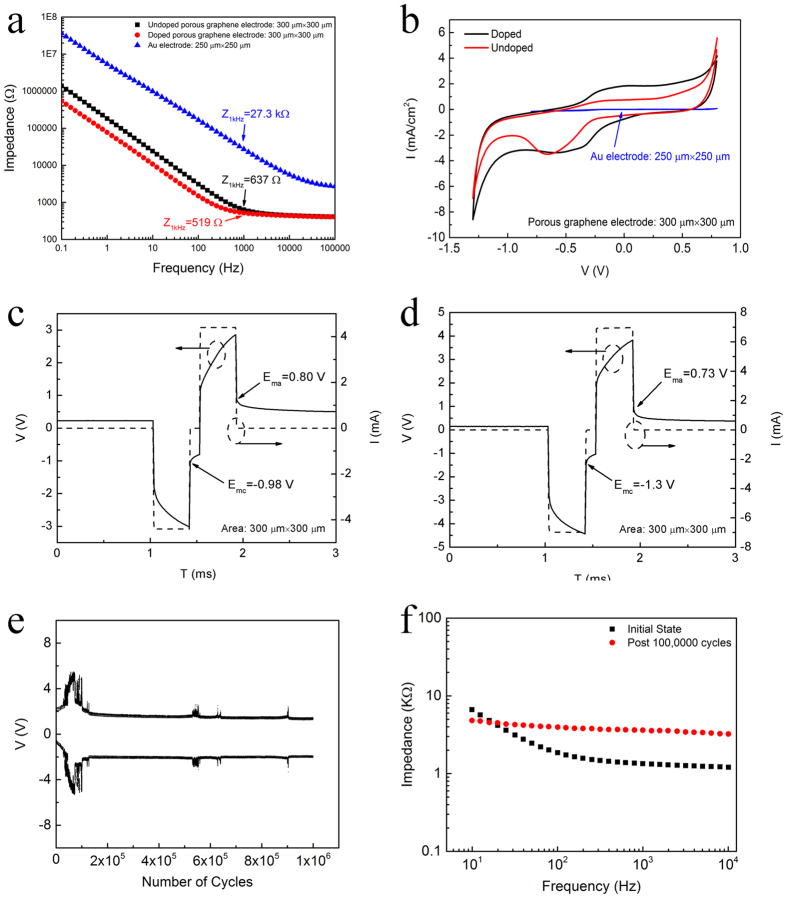
Electrochemical characterization. (**a**) Electrochemical Impedance Spectroscopy (EIS) comparison among doped and undoped porous graphene and Au electrodes. (**b**) CV characterization of porous graphene and Au electrodes. Measurement of the charge injection limit with cathodal-first symmetric biphasic current pulses for the porous graphene electrode (**c**) before and (**d**) after doping. (**e**) Cycling with biphasic stimulation pulses to study degradation during charge injection. (**f**) EIS before and after 1 million stimulation cycles.

Detailed electrochemical measurements were performed on individual electrodes (Gamry Reference 600 Potentiostat). The impedance of a porous graphene electrode (Sample #1) was found to be approximately two orders of magnitude smaller than a similar-size Au electrode. Chemical doping further decreased the impedance, resulting in an impedance of 519 Ω at 1 kHz, as shown in Fig. 3a. Low impedance of the porous graphene electrodes confirms its potential for scaling the electrode dimensions and spatial resolution down to 10 μm. Cyclic voltammetry (CV) measurements were carried out to compare charge storage capacity of porous graphene and Au electrodes (Fig. 3b). While the water window of the Au electrode was −0.8 V to 0.8 V, it was extended to −1.5 V to 0.8 V for the porous graphene electrode, as determined by the sharp increase in oxidation and deoxidization currents. Carbon species usually show a larger water window than metal electrodes, which is essential for exhibiting enhanced charge transfer capacity^21^. The CIC is defined as the maximum quantity of charge that an electrode can inject without reaching beyond the water window, which limits the maximal safe current stimulus. Symmetric biphasic cathodal-first current pulses with 400 ms pulse width and interphase period of 100 ms were applied to the electrodes. Voltage transients were measured to determine the maximum polarization, i.e. the most negative (E_mc_) and most positive (E_ma_) voltages across the electrode/electrolyte interface. Maximum polarization is reached when either E_mc_ or E_ma_ exceeds the water window. Figure 3c displays the voltage transient of an undoped porous graphene electrode, with E_mc_ and E_ma_ reaching −0.98 V and 0.8 V, respectively. The injection current was 4.4 mA. Figure 3d shows the voltage transient of a doped porous graphene electrode, and the injection current increased to 7 mA. Doping helped to increase the CIC from 2 mC/cm^2^ to 3.1 mC/cm^2^. High CIC results were consistent across many samples fabricated at different batches (Figures S2–4). Table S1 in Supplementary Information compares charge storage capacity (CSC_c_) and CIC for neural electrodes made of various materials. Coating layers, such as iridium oxide, poly(3,4-ethylenedioxythiophene) (PEDOT) and carbon nanotubes, have been employed for increasing the electrodes’ surface area and hence charge transfer capability. However, mechanical failures due to cracking and delamination pose a threat to the surrounding tissue, limiting their use for long-term chronic studies or implantable medical systems^17^,^18^. For example, thicker PEDOT coatings have been observed to suffer more cracking and delamination due to the higher stress imposed on the film^18^. A threshold between 2 mC/cm^2^ and 3 mC/cm^2^ CIC for delamination of iridium oxide has been shown in *in vivo* experiments^17^. On the contrary, porous graphene is formed directly by pyrolysis of the bulk polyimide films, providing a substantially stronger adhesion. In this work, all the samples were repeatedly used in charge injection measurements and *in vivo* cortical stimulations, with current injections as high as 3.1 mC/cm^2^. However, no change in impedance or physical appearance of the electrodes was observed. Furthermore, SEM inspections of the porous graphene electrodes after soaking in PBS solution for 30 days show no obvious delamination or physical degradation compared to an unused electrode (Figure S6). Mechanical durability of porous graphene has been previously demonstrated for supercapacitor applications^45^. We tested stimulation performance and cycling endurance by subjecting the porous graphene electrodes to continuous biphasic, cathodal first, charge balanced current pulses with 0.75 mA amplitude and 400 μs durations for cathodic and anodic phases. Figure 3e shows the stable voltage window over 1 million stimulation cycles. Initial fluctuations in the voltage window are attributed to the impedance fluctuations of the porous graphene due to activation/inactivation of defect sites in the fresh sample. No physical degradation of the porous graphene due to charge injection is observed after 1 million cycles. Figure 3f shows that the impedance of the electrode only slightly increased and it was still low enough to allow high charge injection capacity after 1 million stimulation cycles.

**Figure 4:**
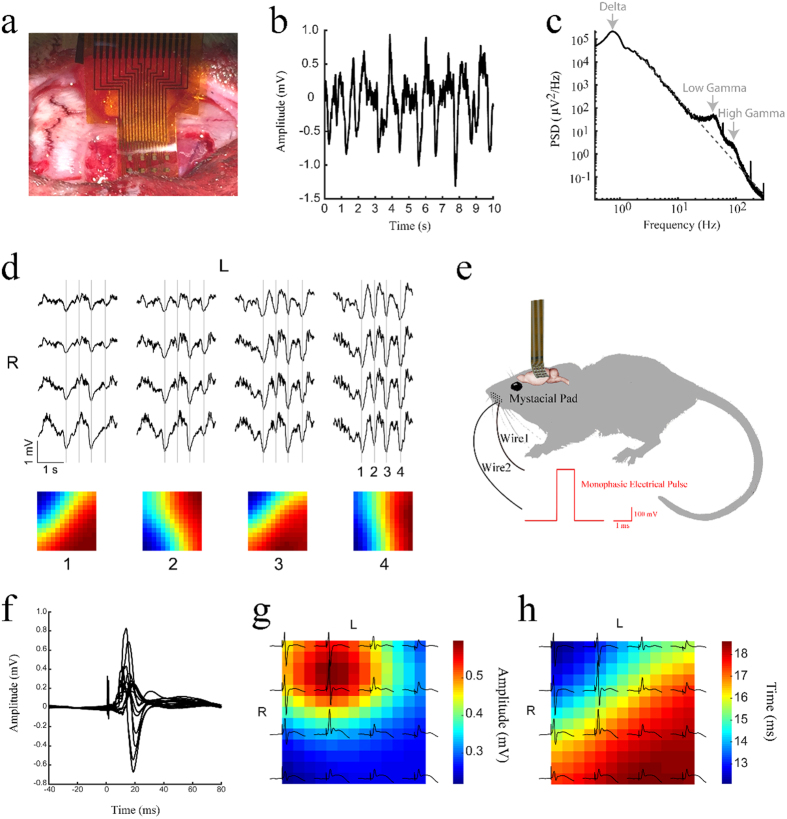
*In vivo* high resolution cortical sensing. (**a**) A 16-electrode array placed at the pial surface of barrel cortex. (**b**) A 10-second recording example from an electrode. (**c**) Average power spectral density of the recorded signal over 5 minutes. (**d**) Spontaneous up and down states recorded cross the 16-electrode array. The distribution of down state amplitudes varied on a cycle-by-cycle basis. (**e**) Schematic of somatosensory-evoked potential recording setup. (**f**) Recorded somatosensory-evoked potentials. (**g**) The amplitude and (**h**) latency of the first positive peak of the somatosensory-evoked potentials.

*In vivo* neural recording experiments were performed on adult rat models. An anaesthetized rat was placed with its head fixed in a stereotaxic apparatus. A craniotomy exposed 4 mm × 4 mm region of right barrel cortex. A 16-electrode array was placed on the exposed cortical surface, as shown in Fig. 4a. Recordings were taken in reference to a distant stainless steel bone screw inserted through the skull during the surgery. In Fig. 4b, a representative example of 10-second recording from one of the electrodes in the array shows spontaneous up and down states of barrel cortex activity, implying active and inactive states of neuronal networks. The average power spectral density computed from the entire 5-minute recording exhibits three prominent oscillations with center frequencies of 0.8 Hz, 40 Hz, and 90 Hz (marked by gray arrows in Fig. 4c). These frequencies correspond to delta, low gamma, and high gamma rhythms, physiological oscillations generated by the brain.

Field potentials at the pial surface of barrel cortex in the anesthetized rat were recorded showing spontaneous up and down states cross the 16-electrode array, as shown in Fig. 4d. Four down cycles, marked by the gray lines, can be seen in the 3-second segment of the recording with each of the 16 electrodes. The distribution of down state amplitudes varies on a cycle-by-cycle basis. The color maps show the relative amplitude interpolated across the array for each of the cycles. In order to assess the capability of recording both spatial and temporal distribution of evoked potentials with porous graphene electrodes, whisker stimulation experiments were performed. A pair of needle electrodes was used to electrically stimulate the left mystacial pad, as illustrated in Fig. 4e. The somatosensory-evoked potentials (SEPs) recorded at the pial surface of barrel cortex by the 16-electrode array are shown in Fig. 4f. The amplitude and latency of the first positive peak of the SEPs varied systematically across the array, shown in Fig. 4g,h, respectively. Similar evoked potential recordings with a 64-electrode array shown in Figure S8 in Supplementary Information.

**Figure 5:**
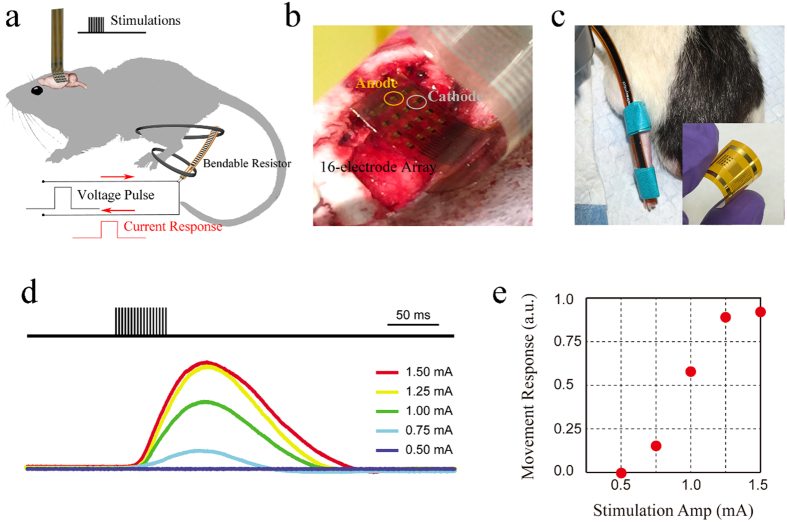
*In vivo* stimulation from cortical surface. (**a**) Schematic of cortical stimulation evoking transient ankle and knee flexion in the hindlimb. (**b**) A 16-electrode array placed on the pial surface of the motor cortex. (**c**) A resistive flex sensor spanning the knee joint. The inset illustrates the flexibility of the 16-electrode array as fabricated. (**d**) Stimulus evoking current (representing movement) response of the flex sensor in arbitrary units. (**e**) Movement response versus stimulation amplitude.

After recording physiological oscillations and evoked potentials, the next step was to investigate cortical microstimulation with porous graphene arrays. Using an array placed over motor cortex this time, stimulus trains were applied to a rat animal model to evoke transient ankle and knee flexion in the contralateral leg, as illustrated in Fig. 5a. Figure 5b demonstrates the placement of the electrode array covering the motor cortex. Knee flexion was only activated when the stimulation was applied on a particular anode electrode overlapping with the leg area in the motor cortex. By changing the stimulation site and scanning across the array, we were able to localize the hindlimb area precisely, suggesting the use of electrical microstimulation with high density porous graphene arrays to map cortical areas with high resolution and precision. The local bipolar stimulus trains were 17 anodic pluses with 0.2 ms pulse width and 3 ms inter-pulse interval. Movement of the hindlimb was measured using a resistive flex sensor spanning the knee joint, as shown in Fig. 5c. A voltage pulse was applied through the sensor, and the change of the corresponding current was measured simultaneously. Amplitude of the stimulus trains ranged from 0.5 mA to 1.5 mA. The higher the stimulus current, the stronger movement was recorded (Video S1, Supplementary Information). No movement was evoked for stimulus less than 0.75 mA (Video S2, Supplementary Information) and saturation of response started at around 1.25 mA as shown in Fig. 5e. We evaluated electrical threshold for stimulation induced tissue damage using Shannon equation, which describes the boundary between tissue damaging and non-damaging levels of electrical stimulation based on empirical data^46^. For *in vivo* cortical stimulation experiments, currents lower than 1.25 mA correspond to k parameters below 1.85, while achieving successful ankle and knee flexion control. Previous experimental studies have also shown that no tissue damage would be induced by small surface electrodes (areas less than 0.01 cm^2^) as long as stimulation is performed within the Shannon limit for k = 1.85^47^. Those findings suggest that porous graphene electrodes can be employed for high-efficiency safe electrical stimulation in therapeutic applications involving modulation of central and peripheral nervous system.

In summary, the flexible porous graphene electrode arrays presented in this paper could be a powerful tool for neuroscience research, particularly for electrical microstimulation, and high density spatio-temporal cortical mapping applications. High CIC and lack of delamination and degradation for porous graphene electrodes can open up new avenues for brain computer interfaces based on minimally invasive cortical stimulation. The elimination of depth electrodes could improve the efficiency of clinical treatments, such as deep brain stimulation for Parkinson’s and responsive neuro-stimulation for epilepsy.

## Methods

### The methods were carried out in “accordance” with the relevant guidelines

#### Fabrication of 3D porous graphene by direct laser pyrolysis method

A laser engraving and cutting system (PLS6.75, Universal Laser Systems Inc.) was used for irradiating polyimide films (50 μm thick, Kapton). CO_2_ laser with wavelength at 10.6 μm was adopted.

#### Raman spectroscopy

Raman spectra of the porous graphene were taken by NTEGRA Spectra (NT-MDT Co.) system with a 532-nm laser excitation source.

#### Porous graphene electrode fabrication

After the patterning of porous graphene, the polyimide film was cleaned with acetone, isopropyl alcohol, and deionized water, followed by de-moisturizing on a hotplate at 150 °C for 5 minutes. Then the polyimide film was attached to a 4 inch Si wafer spin-coated with polydimethylsiloxane (PDMS), which is helpful to maintain the film flat during all following processes. Cr/Au (10 nm/100 nm) layers were deposited with electron-beam evaporation, and the metal wires and contact pads were patterned with S1818 photoresist and wet etching. 9 μm thick SU8-2007 was spin-coated and patterned for encapsulation. Electrode openings were 250 μm × 250 μm. Doping in nitric acid (70%) for 30 seconds helped to decrease the impedance of the electrodes.

#### Electrochemical characterization

The Gamry Reference 600 potentiostat was connected in the standard three-electrode configuration in 0.01 M PBS solution. The counter electrode was Pt and the noncurrent-carrying reference electrode was Ag/AgCl. EIS measurements were taken between 0.1 Hz to 300 kHz using 10 mV RMS AC voltage. For CV tests, the potential of the working electrode swept three times across the water window at the scan rate of 100 mV/s. In choronopotentometry measurements, three successive identical biphasic current pulses were applied after stabilization of the system, and the simultaneous voltage transient was recorded. CSC_c_ is calculated from the time integral of the cathodic current of the CV within the water electrolysis window and divided by the geometry surface area of the electrode. CIC is calculated from the current pulse divided by the geometry surface area of the electrode.

#### *In vivo* neural recording and stimulation

Experiment procedures were approved by the Institutional Care and Use Committee of the University of Pennsylvania. Two rats were used. Each rat was anesthetized with a ketamine (60 mg/kg), dexdomitor (0.25 mg/kg) solution and placed in a stereotaxic frame. The ketamine-dexdomitor solution, at the concentrations we used in the experiments, put the animal at a surgical plane of anesthesia (loss of eyeblink and withdrawal reflexes, 60–80 breathes/min), as required for doing a craniotomy. Craniotomy was performed to expose the right barrel cortex (recording experiments) or motor cortex (stimulation experiment). A skull screw was placed in the left frontal bone to serve as the reference electrode for the recordings. The array was placed on the exposed cortical surface. For recording evoked activities, a pair of needles was used to electrically stimulate the left mystacial pad. Wide-band (0.35–7500 Hz) evoked and spontaneous cortical activity was recorded at 25 kS/s (ZC16, PZ2, RZ2, Tucker-Davis Technologies).

## Additional Information

**How to cite this article**: Lu, Y. *et al*. Flexible Neural Electrode Array Based-on Porous Graphene for Cortical Microstimulation and Sensing. *Sci. Rep.* **6**, 33526; doi: 10.1038/srep33526 (2016).

## Supplementary Material

Supplementary Information

Supplementary Video S1

Supplementary Video S2
