# Supplementary material for: Flexible Neural Electrode Array Based-on Porous Graphene for Cortical Microstimulation and Sensing
Source: Sci Rep. 2016 Sep 19;6:33526. doi: 10.1038/srep33526 (PMC5027596; doi:10.1038/srep33526)
Supplement: Supplementary Information [file srep33526-s1.pdf]

Flexible Neural Electrode Array Based-on Porous Graphene for Cortical Microstimulation and Sensing

Yichen Lu [†]*, Hongming Lyu* [†]*, Andrew G. Richardson, Timothy H. Lucas, and Duygu Kuzum**

Yichen Lu and *Hongming Lyu* contribute equally to this paper.

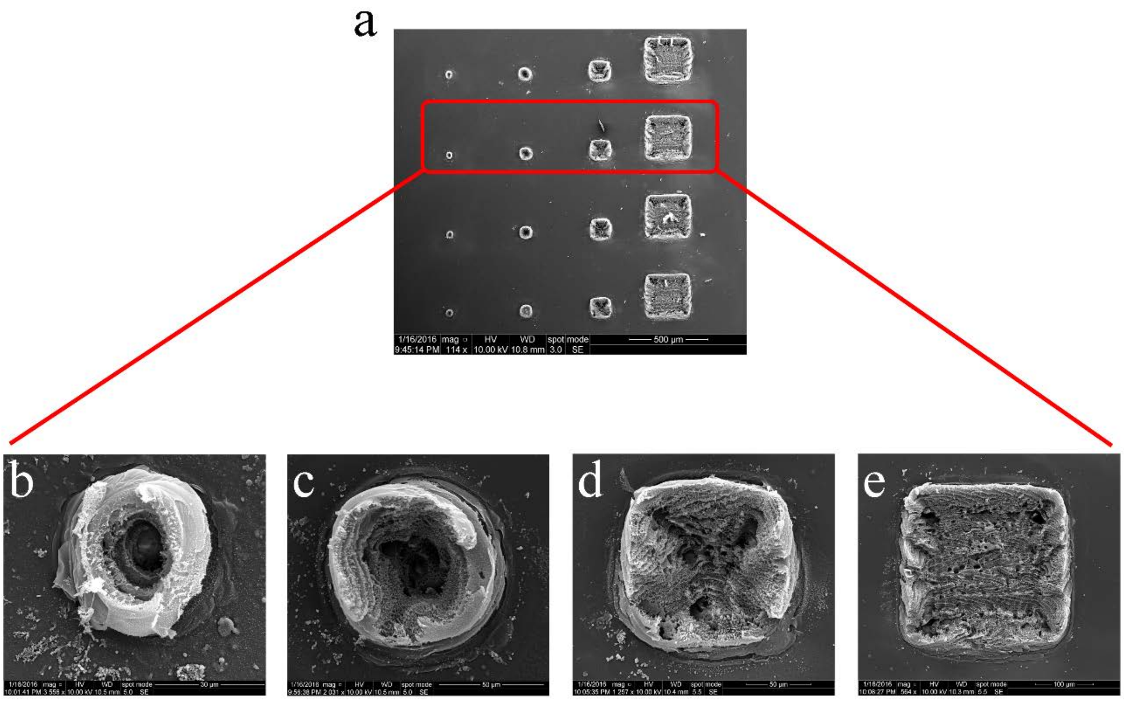

Figure S1. SEM of porous graphene spots printed through shadow masks. a) Overall view of a porous graphene array of spots with difference sizes. b-e) present spots with 25 µm, 50 µm, 100 µm and 250 µm side lengths, respectively. Shadow mask method of laser printing ensures good shape control on spots with sides larger than 100 µm. For 25 µm and 50 µm spots, further shadow mask engineering is needed for better uniformity.

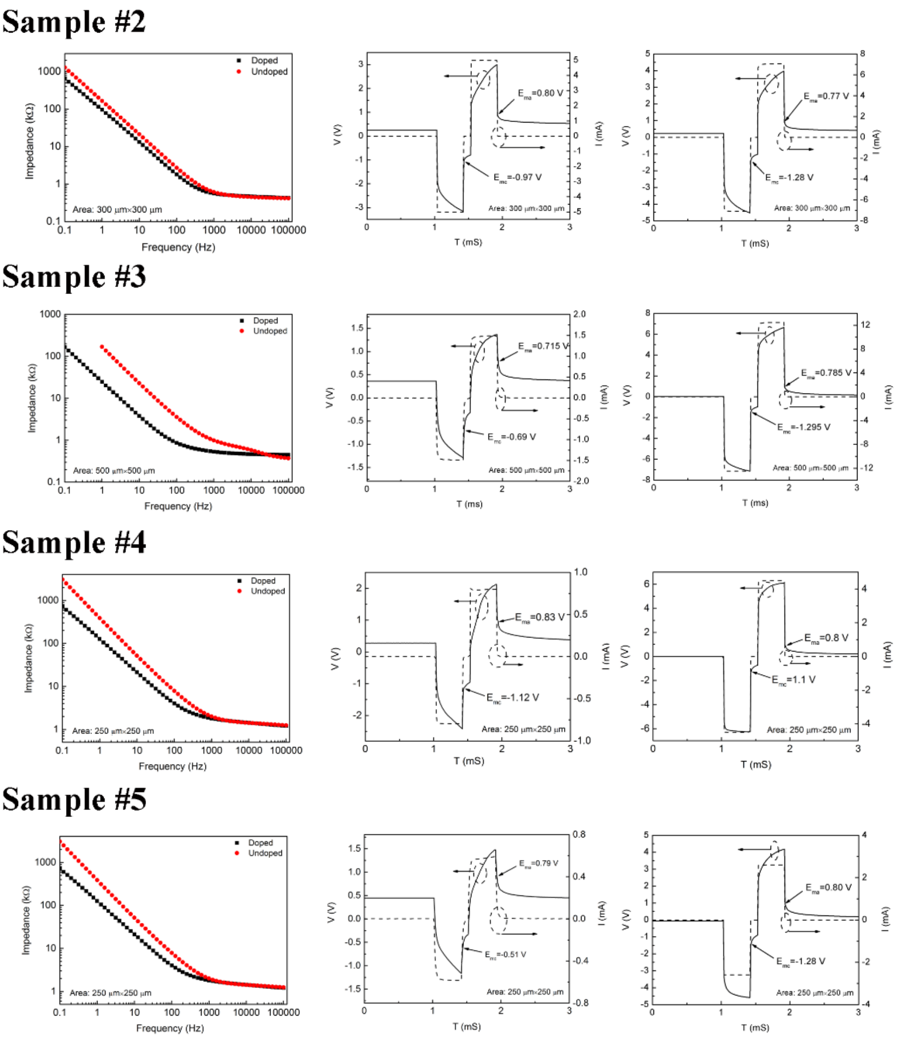

Figure S2. Electrochemistry characterization results of more typical porous graphene electrodes. The first, second, third and fourth rows are sample #2, sample #3, sample #4 and sample #5, respectively. The first, second, and third columns are the EIS results, voltage transients in response to applied current pulses before and after doping, respectively.

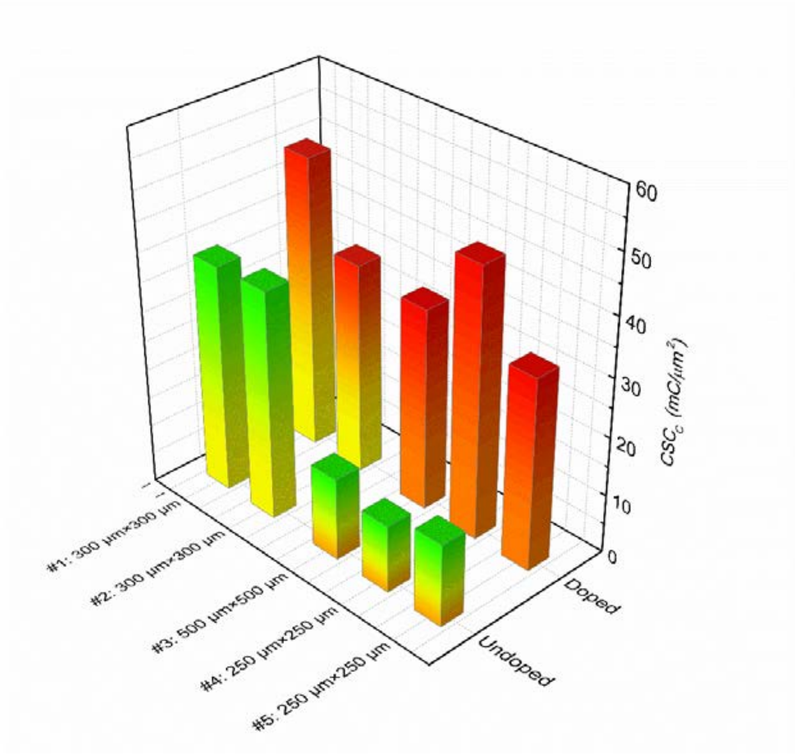

Figure S3. Cathodic charge storage capacity of five representative porous graphene electrodes.

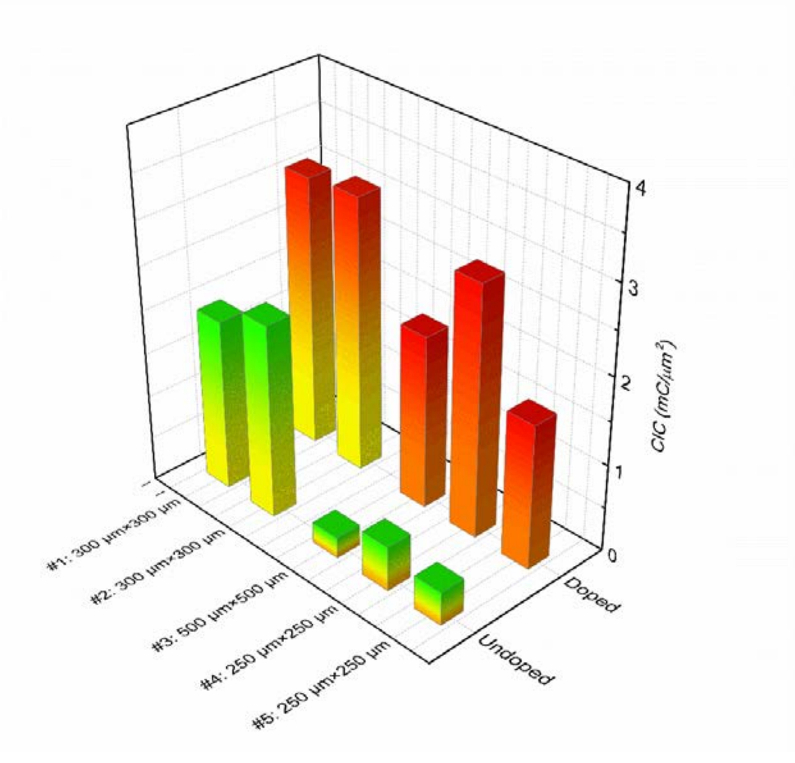

Figure S4. Charge Injection capacity of five representative porous graohene electrodes.

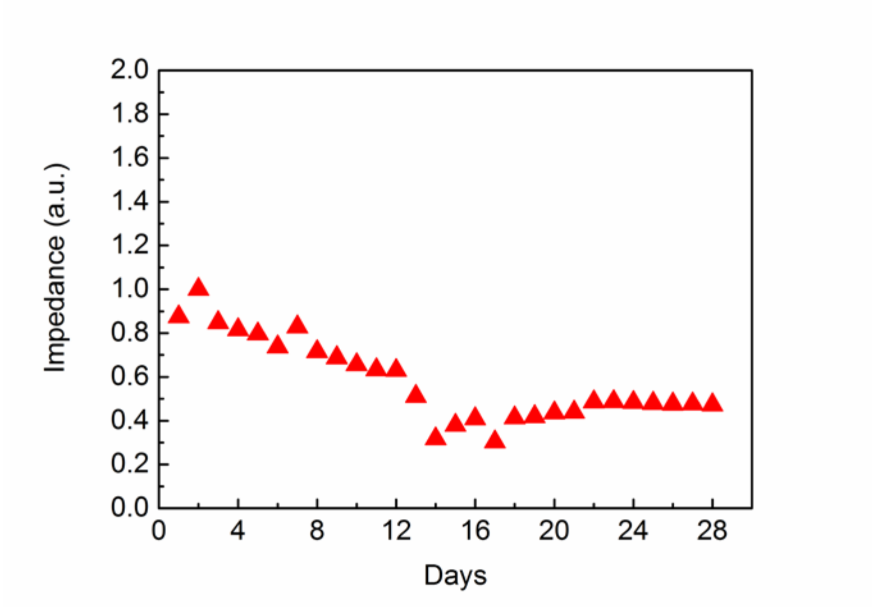

Figure S5. Longevity of doping treatment's impact on impedance over 28 days.

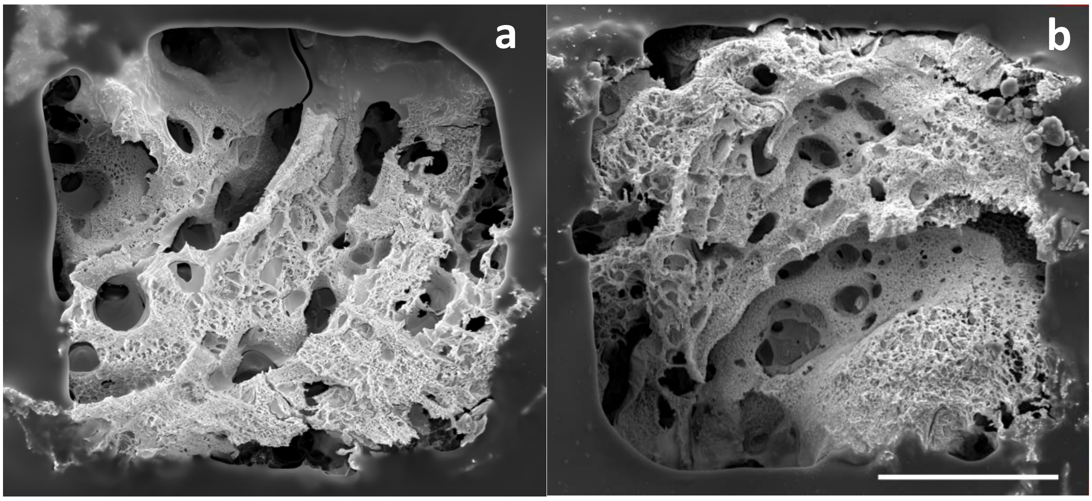

Figure S6. (a) SEM of an unused electrode; (b) SEM image of an porous graphene electrode after using in the animal experiment and soaking in PBS solution for a month. Scale bar: 40 μm

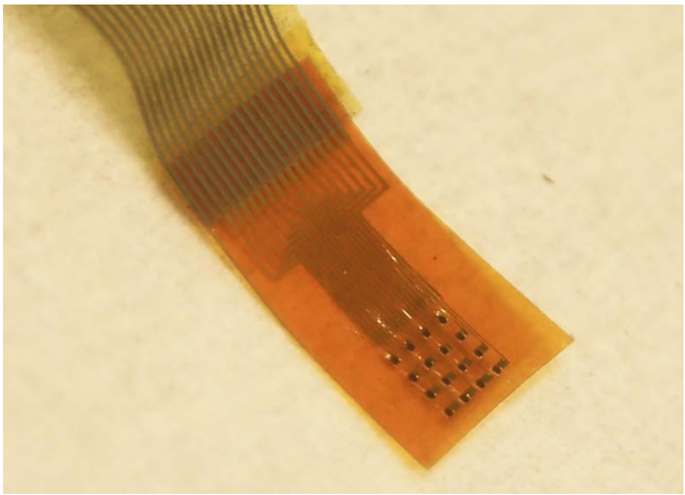

Figure S7. Photograph of a 16-electrode array.

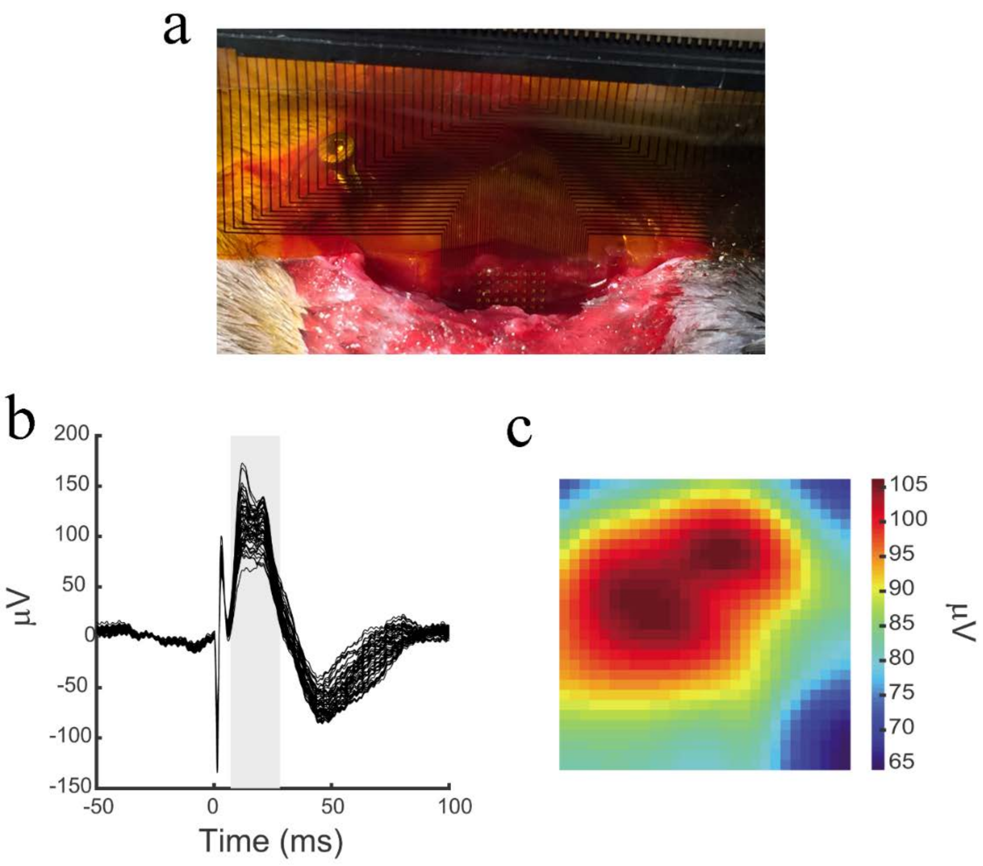

Figure S8. Evoked potential recording with a 64-electrode array. A pair of needle stimulating electrodes were used to electrically stimulate the mystacial pad of a rat model. a) Placement on the pial surface of barrel cortex. b) Recorded evoked potentials. c) The amplitude of the first positive peak of the evoked potentials.

Table S1. Comparison of state-of-art neural electrodes.

Material	Water Window versus Ag/AgCl	CIC (mC/cm^2)	CSC (mC/cm^2)	Ref
PrIr	-0.6 V to 0.8 V	0.15-0.3		[1]
Pt	-0.6 V to 0.8 V	0.05-0.15	0.55 @20mV/s	[2, 4]
TiN	-0.9 V to 0.9 V	0.9	0.25 @20mV/s	[3, 4]
Iridium Oxide	-0.6 V to 0.8 V	2	28.8 @ 50 mV/s	[5, 7]
PEDOT	-0.7 to 0.6 V	2.3	75.6 @ 50 mV/s	[6, 7]
Ta/Ta_2O_5		0.26		[8]
Carbon Nanotube Fiber	-1.5 V to 1.0 V	1-1.6		[9]
Carbon Nanotube Coating	-0.9 V to 0.5 V		5~25 @100 mV/s	[10]
This Work	-1.3 V to 0.8 V	3.2	50 @100 mV/s	

Supplementary Video Legends

Video S1: Video shows that cortical stimulation with current amplitude of 1.25 mA induces ankle and knee flexion. Beep sound represents the timing of stimulation using porous graphene electrodes.

Video S2: Video shows that cortical stimulation with current amplitude of 0.5 mA does not evoke leg movement.

References

[1] L. Bullara, D. McCreery, T. Yuen, W. Agnew, Journal of Neuroscience Methods 1983, 9, 15.
[2] T. Rose, L. Robblee, Biomedical Engineering, IEEE Transactions on 1990, 37, 1118.
[3] J. D. Weiland, D. J. Anderson, M. S. Humayun, Biomedical Engineering, IEEE Transactions on 2002, 49, 1574.
[4] S. F. Cogan, Annu. Rev. Biomed. Eng. 2008, 10, 275.
[5] S. F. Cogan, A. A. Guzelian, W. F. Agnew, T. G. Yuen, D. B. McCreery, Journal of Neuroscience Methods 2004, 137, 141.
[6] X. T. Cui, D. D. Zhou, Neural Systems and Rehabilitation Engineering, IEEE Transactions on 2007, 15, 502.
[7] S. J. Wilks, S. M. Richardson-Burns, J. L. Hendricks, D. C. Martin, K. J. Otto, Frontiers in Neuroengineering 2009, 2, 7.
[8] T. Rose, E. Kelliher, L. Robblee, Journal of Neuroscience Methods 1985, 12, 181.
[9] K. Wang, H. A. Fishman, H. Dai, J. S. Harris, Nano Letters 2006, 6, 2043.
[10] E. Jan, J. L. Hendricks, V. Husaini, S. M. Richardson-Burns, A. Sereno, D. C. Martin, N. A. Kotov, Nano Letters 2009, 9, 4012.
